# The Omics‐Driven Machine Learning Path to Cost‐Effective Precision Medicine in Chronic Kidney Disease

**DOI:** 10.1002/pmic.202400108

**Published:** 2025-01-10

**Authors:** Marta B. Lopes, Roberta Coletti, Flore Duranton, Griet Glorieux, Mayra Alejandra Jaimes Campos, Julie Klein, Matthias Ley, Paul Perco, Alexia Sampri, Aviad Tur‐Sinai

**Affiliations:** ^1^ Center for Mathematics and Applications (NOVA Math) NOVA School of Science and Technology (NOVA FCT) Caparica Portugal; ^2^ UNIDEMI, Department of Mechanical and Industrial Engineering NOVA School of Science and Technology (NOVA FCT) Caparica Portugal; ^3^ RD‐Néphrologie Montpellier France; ^4^ Department of Internal Medicine and Pediatrics Nephrology Unit Ghent University Hospital Gent Belgium; ^5^ Department of Biomarker Research Mosaiques Diagnostics GmbH Hannover Germany; ^6^ Institute for Molecular Cardiovascular Research University Hospital RWTH Aachen Aachen Germany; ^7^ Institut National de la Santé et de la Recherche Médicale (INSERM) Institute of Cardiovascular and Metabolic Disease Toulouse France; ^8^ Université Toulouse III Paul‐Sabatier Toulouse France; ^9^ Delta4 GmbH Vienna Austria; ^10^ Division of Pediatric Nephrology and Gastroenterology Department of Pediatrics and Adolescent Medicine Comprehensive Center for Pediatrics Medical University Vienna Vienna Austria; ^11^ Department of Internal Medicine IV Medical University Innsbruck Innsbruck Austria; ^12^ Department of Public Health and Primary Care British Heart Foundation Cardiovascular Epidemiology Unit University of Cambridge Cambridge UK; ^13^ Victor Phillip Dahdaleh Heart and Lung Research Institute University of Cambridge Cambridge UK; ^14^ School of Public Health University of Haifa Haifa Israel

**Keywords:** artificial intelligence, chronic kidney disease, cost‐effectiveness, multi‐omics, machine learning

## Abstract

Chronic kidney disease (CKD) poses a significant and growing global health challenge, making early detection and slowing disease progression essential for improving patient outcomes. Traditional diagnostic methods such as glomerular filtration rate and proteinuria are insufficient to capture the complexity of CKD. In contrast, omics technologies have shed light on the molecular mechanisms of CKD, helping to identify biomarkers for disease assessment and management. Artificial intelligence (AI) and machine learning (ML) could transform CKD care, enabling biomarker discovery for early diagnosis and risk prediction, and personalized treatment. By integrating multi‐omics datasets, AI can provide real‐time, patient‐specific insights, improve decision support, and optimize cost efficiency by early detection and avoidance of unnecessary treatments. Multidisciplinary collaborations and sophisticated ML methods are essential to advance diagnostic and therapeutic strategies in CKD. This review presents a comprehensive overview of the pipeline for translating CKD omics data into personalized treatment, covering recent advances in omics research, the role of ML in CKD, and the critical need for clinical validation of AI‐driven discoveries to ensure their efficacy, relevance, and cost‐effectiveness in patient care.

**Abbreviations**
CKD: chronic kidney disease
CNN: Convolutional Neural Network
CVD: cardiovascular disease
DKD: diabetic kidney disease
DL: deep learning
DN: diabetic nephropathy
eGFR: estimated glomerular filtration rate
eQTL: expression quantitative trait loci
GFR: glomerular filtration rate
ICM: ischemic cardiomyopathy
K‐NN: K‐nearest neighbors
LDA: linear discriminant analysis
LR: logistic regression
ML: machine learning
MLP: multilayer perceptron
MR: mendelian randomization
PLS‐DA: partial least squares—discriminant analysis
PPI: protein‐protein interactions
RF: random forest
SNP: single nucleotide polymorphisms
SVM: support vector machines
SVM‐RFE: support vector machines‐feature recursive elimination
T2D: type 2 diabetes
t‐SNE: t‐distributed stochastic neighbor embedding
UMAP: uniform manifold approximation and projection

## 1 Introduction

Chronic Kidney Disease (CKD) represents a critical challenge for public health, affecting approximately 10% of the global population and is estimated to become the 5th leading cause of death worldwide by 2050 [1]. Diabetes and hypertension are the main causes of CKD [2]. Among patients with CKD, cardiovascular disease (CVD), accounts for nearly 50% of all deaths compared to 26% in the population with normal kidney function [3]. CKD is characterized by a gradual decline in kidney function. In the early stages, symptoms are unclear which often makes the disease go unnoticed. As the disease progresses, patients may experience a range of non‐specific symptoms. Ultimately, if left untreated, CKD will lead to irreversible kidney damage, necessitating expensive kidney replacement therapies (KRT) such as dialysis. This brings a considerable burden on healthcare systems and the environment but also significantly impacts patients' quality of life [4].

Classification of CKD is based on cause, glomerular filtration rate (GFR) category, and albuminuria category [5]. However, they often fail to capture the complexity of diverse outcomes and variations in disease progression and treatment responses. In contrast, omics approaches offer a comprehensive analysis of genes, gene transcripts, proteins and metabolites, generating big data. This shift has greatly increased our understanding of CKD's underlying mechanisms and led to the development of biomarkers for improved risk assessment [6].

At the end of 2023, the Partnership for Health System Sustainability and Resilience (PHSSR) European Union (EU) expert advisory group published policy recommendations to enhance the prevention and early detection of non‐communicable diseases, including CKD and CVD [7]. Implementing these recommendations requires significant efforts in identifying and validating biomarkers for high‐risk individuals, facilitating early diagnosis and risk stratification.

Artificial Intelligence (AI), particularly Machine learning (ML), is essential in deepening our understanding of these diseases, speeding up drug discovery, and facilitating drug repurposing. ML algorithms and big data resources, particularly omics data, have the potential to uncover novel biomarkers for early CKD detection and monitoring. While previous review articles have discussed the potential of big data and ML in nephrology, most focus on electronic health records (EHR) or provide a broad overview of kidney diseases. Few reviews address insights from omics studies in nephrology and kidney disease with particularly limited coverage of CKD [8, 9, 10, 11, 12].

**FIGURE 1:**
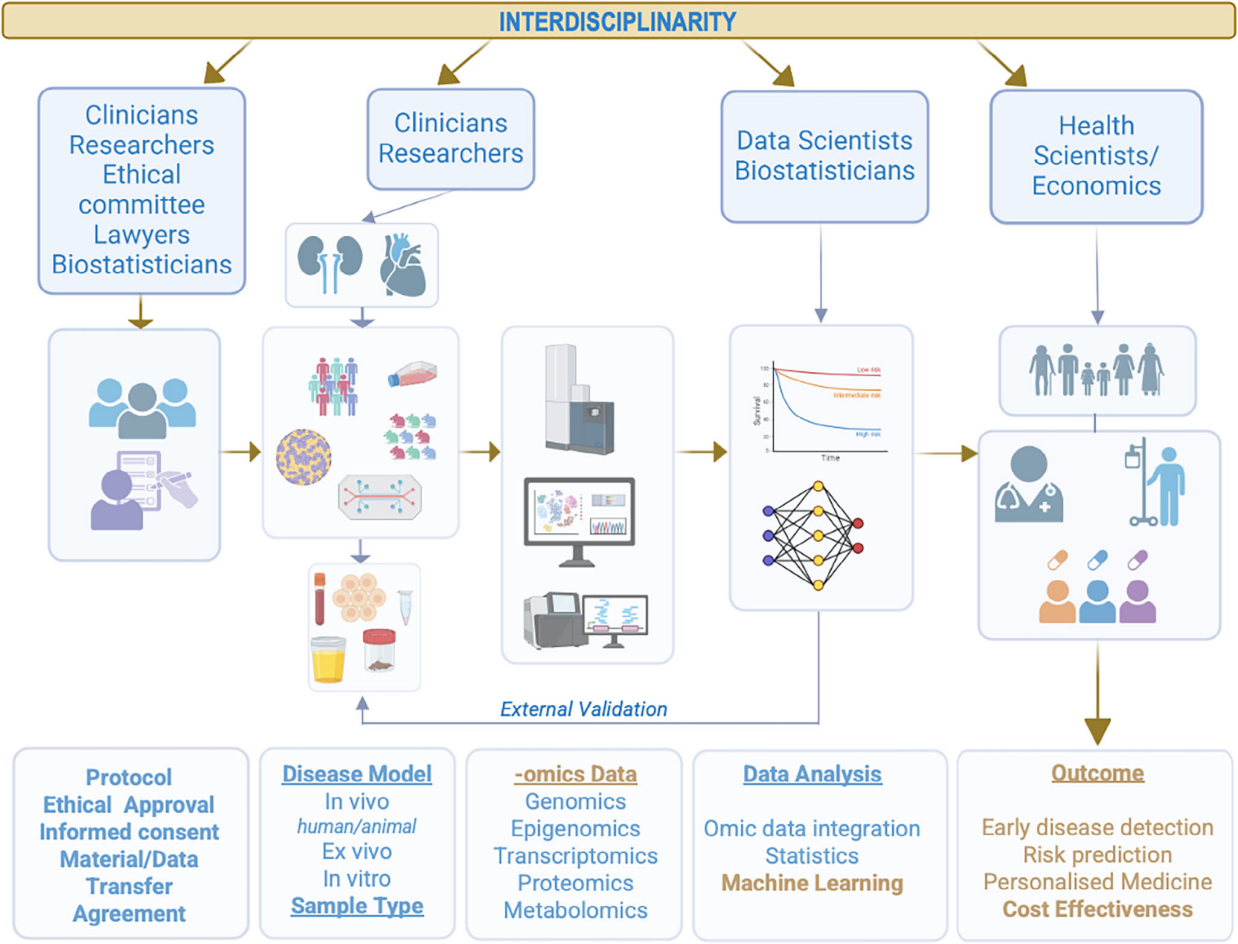
The multi‐omics ML pipeline in chronic kidney disease (CKD). Achieving early disease detection, risk prediction, and personalized medicine in CKD requires an interdisciplinary approach. Integrating available omics datasets from various experimental and clinical studies is essential. ML is promising to extract insights from complex omics datasets, but external validation is necessary to ensure clinical relevance and assess cost‐effectiveness. Optimization of the entire pipeline (data availability, standardized disease, and preclinical and clinical validation) is of utmost importance to allow developing novel tools and improving patient outcomes in CKD. Created with BioRender.com.

This review examines the unique characteristics of various omic layers, key consortia supporting CKD omics research, and the evolving landscape of ML for deriving insights from complex omics datasets. Additionally, we emphasize the need for rigorous preclinical and clinical validation of ML findings to ensure their clinical relevance and the development of models to estimate the cost‐effectiveness of AI‐driven discoveries in clinical practice. These combined multidisciplinary efforts are essential steps toward achieving personalized medicine in CKD (Figure 1).

## 2 The Promise of Omics in CKD Clinics and Research

### 2.1 The Omics Landscape of CKD

Omics data have significantly advanced our understanding of the biological mechanisms driving the high heterogeneity of CKD. Here, we summarize key insights across various omics studies conducted in CKD patients.

#### 2.1.1 Genetic Polymorphisms

Genome‐wide association studies have identified genetic polymorphisms linked to CKD, particularly single nucleotide polymorphisms (SNPs), with ancestry‐dependent variations [13]. Genes such as non‐muscle myosin heavy chain 9 (*MYH9*), apolipoprotein L1 (*APOL1*), apolipoprotein E (*APOE*), SPARC‐related modular calcium‐binding 2 (*SMOC2*), Telomerase reverse transcriptase (*TERT*), collagen type IV (*COL4*), and uromodulin (*UMOD*), among others, have been implicated in CKD risk across diverse population [14]. Moreover, recent Mendelian randomization (MR) studies have linked genetic variants to CKD onset and progression, identifying causal risk factors [15]. Expression quantitative trait loci (eQTL) analyses have further highlighted novel targets, including lysosomal β‐glucosidase, *TGF‐β*, and *DAB2* [16, 17].

#### 2.1.2 Epigenomics

Epigenetic mechanisms such as DNA methylation, histone modifications, and non‐coding RNAs play vital roles in CKD, mediating genetic‐environment interactions [18, 19]. The most well‐known epigenetic marks include DNA methylation of cytosines, post‐translational modifications (PTMs) of histones, and non‐coding RNAs (ncRNA) [20, 21]. Current findings suggest that methylation risk scores could improve CKD classification and predict disease progression [22, 23], while studies show that diabetic kidney disease (DKD) involves hyperglycemia‐induced epigenetic modifications affecting mitochondrial function and immune activation [23].

#### 2.1.3 Transcriptomics

Transcriptomics studies in CKD and more specifically diabetic nephropathy (DN) have focused on messenger RNA (mRNA), microRNA (miRNA) [24, 25], and different ncRNA [26, 27, 28]. Advances from microarrays and bulk RNA‐seq to single‐cell RNA‐seq (scRNA‐seq) and spatial transcriptomics, have enabled the identification of key kidney cell types and their responses to injury [29, 30]. Recently, a scRNA‐seq study explored kidney‐protective mechanisms, identifying target cells of mineralocorticoid receptor antagonists [31]. Another recent study demonstrated that SGLT2 inhibitors benefit DKD patients by suppressing mTORC1 signaling in kidney tubules, thereby reducing transcript levels in key metabolic pathways [32].

#### 2.1.4 Proteomics and Metabolomics

Classic CKD metabolic markers including urea, creatinine, and uric acid, as well as proteins such as albumin, and cystatin C, remain essential in clinics, while emerging proteomic and metabolomic biomarkers have been proposed for implementation into clinical practice [33, 34]. Novel proteomic biomarkers, including retinol‐binding protein 4, alpha‐1 anti‐chymotrypsin, apolipoprotein C‐III, apolipoprotein L1, haptoglobin, and vitronectin, have been associated with kidney function and the diagnosis of CKD [35, 36]. Recent proteome‐wide MR studies have identified novel therapeutic targets for CKD across diverse ancestral groups and 21 blood proteins as potential therapeutic targets for DKD [37, 38]. Futhermore, Si et al. identified 32 potential CKD drug targets by integrating proteome and transcriptome data, validating 20 novel causal proteins, including *GCKR*, *IGFBP‐5*, *sRAGE*, *GNPTG*, and *YOD1* [39].

Moreover, recent metabolomic analyses have shown a global suppression of mitochondrial activity in DKD and DN, along with the identification of disease‐specific patterns associated with the diagnosis and early detection of these conditions [40, 41]. Metabolites such as 3‐hydroxyisobutyrate (3‐HIBA) and 3‐methylcrotonylglycine were significantly negatively associated with eGFR slope, while citric and aconitic acid were positively associated, even after adjusting for clinical variables [42]. Additionally, pantothenic acid (PA) and the CoA biosynthesis pathway were identified as biomarkers for early DKD detection and progression [43].

#### 2.1.5 Spatial Omics

Recent advances in spatial multi‐omics technologies have revealed novel insights into the intricate interplay of molecular, cellular, and tissue‐level mechanisms driving CKD. Abedini et al. utilized spatial transcriptomics to study CKD, uncovering localized pathways of fibrosis and inflammation that are pivotal to disease progression [44]. Similarly, spatial proteomic, epigenomic, and metabolomic approaches have revealed molecular heterogeneity across distinct anatomical regions, identifying pleckstrin‐homology‐domain‐containing A1 (*PLEKHA1*) as a potential biomarker associated with CKD development [45]. Moreover, a spatial multi‐omics study of long‐standing DKD identified regionally distributed molecular clusters and cell‐specific responses, including differentially expressed lipid metabolites in the inner medullary regions [46].

### 2.2 Open and Collaborative Research in CKD

The rapid expansion of omics technologies has generated vast amounts of experimental data, much of which remains underutilized despite their potential. This observation underscores the need for accessible, well‐curated databases to support efficient data storage, management, and sharing. In CKD, kidney‐specific omics databases provide valuable insights into disease mechanisms and potential molecular targets, significantly advancing medical research [9, 47, 48]. Data sharing not only reduces redundant analyses and associated costs but also facilitates the study of complex datasets derived from otherwise challenging samples, such as invasive kidney biopsies.

The recently published atlas of healthy and injured human kidneys combines transcriptomic, epigenomics, and 3D imaging data from various kidney biopsy biobanks and databases [29]. It relies on the Kidney Precision Medicine Project (KPMP), which provides open access to standard and multi‐omics data from kidney biopsies [26, 49, 50]. Other significant multi‐omics sources include the Human BioMolecular Atlas Program (HuBMAP) and the Human Cell Atlas (HCA) although its kidney atlas is not yet publicly accessible [51, 52]. Several web applications allow for the analysis of kidney‐specific transcriptomics datasets at varying levels of complexity and flexibility, such as the Nephroseq platform the NEPTUNE‐Study NephQTL, Human Kidney eQTL Atlas NephroCell and Kidney Interactive Transcriptomics [53, 54, 55, 56, 57, 58]. More recently, the NIH‐NIDDK Atlas‐D2K platform was built to integrate kidney and genito‐urinary tract histology and transcriptomics data from the GUDMAP and ReBuilding a Kidney (RBK) consortia [59].

Genetic associations with kidney function have been explored using public datasets such as CKDGen PheWeb and the UK Biobank [55, 60, 61, 62, 63]. While no blood or urine CKD‐specific proteomics databases were identified, the MassIVE repository and the preclinical Kidney omics database may provide valuable resources [64, 65]. The latter, in particular, contains a database of 1160 urinary exosome proteins from healthy volunteers [66, 67]. The Extracellular Vesicles miRNA Database (EVmiRNA; [68]) gathers miRNA profiling from different sources of exosomes and microvesicles including urine. Urinary exosomes are interesting material for CKD studies, as they directly originate from urogenital cells [69].

The PRIME‐CKD Consortium [56] (prime‐ckd.com) aims to promote clinical research in CKD by validating biomarkers and developing procedures for personalized medicine in CKD. The Biomarker Enterprise to Attack Diabetic Kidney Disease consortium (BEAt‐DKD) personalized medicine and omics approaches to prevent diabetes, the leading cause of CKD, while the PRIORITY Study addressed DKD prevention [70, 71, 72]. The recent SIGNAL project (Body fluid proteome SIGnatures for persoNALised intervention to prevent cardiovascular and renal complications in diabetes) seeks to identify proteomic signatures for personalized interventions in diabetes‐related complications.

Papadopoulos et al. listed various omics data repositories, noting that only Nephroseq remains active among seven kidney‐specific initiatives, while RGED is under updates [48, 73]. General omics repositories continue to function, except for the Multi‐Omics Profiling Expression Database (MOPED), highlighting that larger repositories are better suited for long‐term data preservation [9, 48].

## 3 Statistical and Machine Learning Approaches for CKD

This section outlines key trends in applying statistics and ML to CKD omics data, addressing CKD's unique challenges while raising awareness in the CKD community of the clinical benefits these advancements offer.

### 3.1 Supervised Learning

Supervised learning, especially classification and regression, is key in predicting CKD‐related phenotypes, stages, and conditions like T2D, DKD, and DN. Various methods, from linear models to advanced non‐linear approaches, are summarized in Table 1.

**TABLE 1:** Summary of studies reporting the use of supervised learning algorithms for the prediction of CKD‐related conditions.

Method	Variable type	Sample type	Outcome variable	Reference
LR	Proteomics	Urine	DKD vs Diabetes; DKD stages	[74]
PCA, PLS‐DA, LR	Metabolomics	Plasma Urine	CKD vs healthy	[75]
K‐NN, PCA, SVM, LR, decision trees	Proteomics Metabolomics Lipidomics	Plasma	Fast eGFR progression vs stable eGFR course	[76]
SVM	Proteomics	Urine	Kidney disease vs healthy	[77]
SVM, Cox regression	Proteomics	Urine	CKD stages; CV events	[6]
Priority‐Lasso, multivariate LR, SVM, RF, AdaBoost	Metabolomics	Serum	CKD vs non‐CKD	[6, 80]
Lasso, RF, SVM‐RFE	Transcriptomics	Kidney Tissue	CKD vs healthy	[111]
Lasso, RF	Proteomics	Urine	T2D vs healthy	[82]
LDA, SVM, RF, LR, PLS‐DA	Proteomics Metabolomics	Serum	DKD vs health; DKD stages	[83]
K‐NN, PCA, SVM, LR, decision trees	Proteomics	Plasma Urine	CKD vs healthy; Different CKD conditions	[84]
Lasso, RF, Naïve Bayes, K‐NN, Extreme Gradient Boosting	Single‐Cell Transcriptomics	Heart Tissue and CKD Peripheral Blood Mononuclear Cells	CKD‐related ICM	[85]
DL, LR, RF, SVM	Metabolomics	Plasma Urine	Rapid eGFR decliners vs. non‐rapid eGFR decliners	[86]
MLP, CNN, DDA, multinomial LR, Naïve Bayes, RF, SVM	Metabolomics	n.r.	CKD stages and healthy	[88]
Lasso, LR, hierarchical clustering, PCA, RF, SVM, multinomial LR, MLP, CNN	Metabolomics	Serum	CKD stages vs healthy	[87]

Abbreviations: LR, logistic regression; PCA, principal component analysis; PLS‐DA, partial least squares‐discriminant analysis; K‐NN, K‐nearest neighbors; SVM, support vector machine; SVM‐RFE, support vector machine‐recursive feature elimination; RF, random forest; DL, deep learning; MLP, multilayer perceptron; CNN, convolutional neural network; n.r., not reported.

Logistic regression (LR) is widely used for supervised classification due to its simplicity and interpretability. Fan et al. applied LR models to urine proteomics data to differentiate between CKD types, while identifying 5‐MTP, homocysteine, and citrulline as CKD biomarkers from plasma and urine metabolic data [74, 75]. Kammer et al. applied Bayesian LR to a multi‐omics DKD dataset to predict eGFR decline, finding KIM‐1 and NTproBNP as key predictors [76].

Good et al. developed the CKD273 classifier, a Support Vector Machine (SVM) model based on a 273‐peptide urine panel, to distinguish kidney disease from controls, validated in multiple following studies [6, 77, 78, 79].

Numerous CKD studies have compared ML algorithms on omics data for disease prediction and staging. Huang et al. used SVM, Random Forest (RF), and AdaBoost on serum metabolomics, identifying sphingomyelin C18:1 and phosphatidylcholine diacyl C38:0 as biomarkers in CKD in prediabetes or T2D patients [80]. Zhong et al. analyzed seven DKD transcriptomics datasets with the least absolute shrinkage and selection operator (lasso) LR, RF, and SVM‐Recursive Feature Elimination (SVM‐RFE), disclosing *TNC*, *PXDN*, *TIMP1*, and *TPM1* as markers linked to oxidative stress and inflammation [81]. Similarly, Yan et al. used lasso and RF on proteomics to highlight KLK1, CSPG4, PLAU, SERPINA3, and ALB as urine biomarkers of DN [82]. Liu et al. applied five ML models (Linear Discriminant Analysis (LDA), SVM, RF, LR, Partial Least Squares—Discriminant Analysis (PLS‐DA)) on serum metabolomics and proteomics, distinguishing DKD from healthy controls and identifying α2‐macroglobulin, cathepsin D, CD324, and glycerol‐3‐galactoside as biomarkers [83].

Glazyrin et al. found K‐Nearest Neighbors (K‐NN) effective in differentiating CKD conditions from plasma and urine proteomics data [84], while Naïve Bayes outperformed other models for identifying CKD‐related ischemic cardiomyopathy (ICM) [85].

Deep learning (DL) has emerged as a powerful tool for analyzing high‐dimensional, non‐linear, and heterogeneous multi‐omics datasets. Hirakawa et al. applied DL on plasma and urine metabolomics to predict eGFR change rates, outperforming LR, RM, and SVM, and identifying biomarkers like systolic blood pressure, albumin‐to‐creatinine ratio, and six metabolites, including urinary 1‐methylpyridin‐1‐ium (NMP) [86]. Comparisons showed that Multilayer Perceptrons (MLP), and RF achieved the highest accuracy for CKD stage classification based on metabolomics data [87, 88].

### 3.2 Unsupervised Learning

Unsupervised learning uncovers hidden patterns in high‐dimensional, unlabeled omics data, which is especially useful when disease classifications are unclear. In CKD studies, Eoli et al. applied nonnegative matrix factorization (NMF), identifying nine clusters related to kidney function, T2D, and body weight [89]. Wilson et al. used canonical correlation analysis and clustering on single‐nucleus RNA‐seq data from human DN, discovering early kidney responses, such as increased potassium secretion, decreased paracellular calcium and magnesium reabsorption, and angiogenic signaling [90]. Reznichenko et al. employed Self‐Organizing Maps and hierarchical clustering on transcriptomics, identifying four distinct molecular disease categories and a 25‐protein signature [57].

Other unsupervised methods include dimensionality reduction techniques, such as Principal Component Analysis (PCA), t‐distributed Stochastic Neighbor Embedding (t‐SNE), and Uniform Manifold Approximation and Projection (UMAP), which reveal hidden structures and aid in visualization. PCA, a linear method, reduces complexity by identifying components that capture maximum variance, while UMAP and t‐SNE, both non‐linear, preserve local and global structures. PCA has been widely applied to omics data to identify CKD‐related groups, (e.g., [75, 84, 87] while UMAP and t‐SNE have also been applied to single‐cell transcriptomics data [29, 82]. The resulting latent representations were then input to clustering algorithms like k‐means and Density‐Based Spatial Clustering of Applications with Noise (DBSCAN), which partition data based on distance or density, respectively.

### 3.3 Multi‐Omics Data Integration

Combining multi‐omics data from high‐throughput technologies is essential for unraveling complex biological mechanisms that single‐omics methods fail to explain. This integration results in significant data heterogeneity and high dimensionality, often with small sample sizes, which pose statistical and computational challenges. To address these challenges, matrix decomposition‐based methods, particularly DIABLO Singh et al., uncover latent factors that represent key biological variations across various omics datasets [91]. DIABLO has identified mechanisms and biomarkers in various disease conditions including T2D, a CKD risk factor, where it showed strong predictive performance when integrating RNA‐seq, DNA methylation, SNPs, and phenotypic data [92, 93, 94, 95].

Another sparsity‐inducing method accounting for multiple data, Priority‐Lasso, prioritizes certain variable groups (e.g., clinical or omics data) in model fitting. High priority is often given to easily or inexpensively collected data, especially in clinical settings where certain types are routinely available. Huang et al. used Priority‐Lasso as part of a three‐step feature selection procedure before a classification task using several ML models to predict CKD incidence [80].

### 3.4 Network Discovery and Analysis

Network‐based algorithms can facilitate the modeling and analysis of vast, highly diverse biomedical knowledge by representing data as interconnected graphs, where nodes correspond to entities, such as genes or diseases, and edges capture their relationships. Resources like PPI networks (e.g., STRING) and GO pathways are essential for CKD research, providing validation for findings and serving as prior knowledge for ML [39, 96]. These networks can be directly analyzed to extract findings, as performed by Masoudi‐Sobhanzadeh et al. who detected key nodes of CKD PPI networks by an optimization approach [97].

Correlation methods effectively identify pairwise associations from omics data. Ahmed et al. used Mutual Information to analyze gene networks in DKD, uncovering clusters of dysregulated genes in immune pathways, consistent with prior research on inflammation's role in DKD [98]. Langfelder and Horvath developed the Weighted Correlation Network Analysis (WGCNA) R package, which offers a collection of correlation‐based methods for gene expression network inference and analysis [99]. This package has been widely employed in CKD studies (e.g., [81, 100, 101]) performing differential gene expression (DEG) analysis, consensus clustering for gene module identification, and network inference. The analysis of these networks has successfully identified biomarkers of CKD progression through methods like lasso, SVM‐RFE, and RF, all integrated within the WGCNA package.

While advancements in network inference have led to more sophisticated methods, their application to CKD remains limited [102]. The graphical lasso (glasso) method infers sparse networks from data, estimating a precision matrix whose entries are zero if the corresponding variables are conditionally independent given the others in the network [103]. Ma et al. extended Glasso method to jointly estimate networks of different groups of CKD patients from lipidomics data, identifying subnetworks that differentiate CKD progressors from non‐progressors [104]. A similar methodological approach was proposed by Danaher et al. with the Joint Graphical Lasso method, estimating sparse networks across patient subgroups using dual penalties [105].

When integrating diverse data types, mixed graphical models can be employed. Altenbuchinger et al. combined metabolomics with clinical data to identify associations with CKD comorbidities [106].

These methods elucidate biological associations, but they do not imply causation. Undirected networks can provide a foundation for discovering causal relationships through complementary methodologies [107], while Bayesian approaches and MR studies can infer causality directly [108].

**FIGURE 2:**
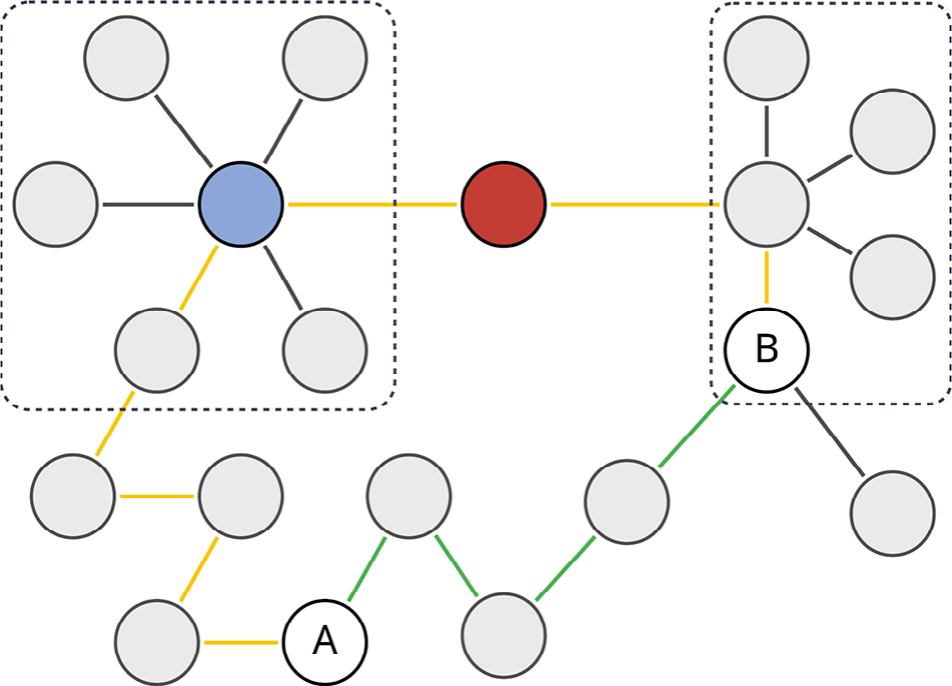
Hypothetical graph illustrating key topological features of a network. The blue node indicates a high degree of centrality, while the red node represents high betweenness centrality. Given a disease‐associated node A, and a predicted target B, the shortest path between nodes A and B is highlighted in green, while the longest path is shown in yellow. The dashed purple lines indicate sample clusters.

After deriving a network, analyzing relationships is essential for insights. Centrality measures, such as closeness and betweenness, alongside community detection methods like the Leiden algorithm, may identify key nodes as potential drug targets [109] or clusters of biologically meaningful nodes [110]. Pathway identification reveals significant biological pathways enriched with differentially expressed genes, supported by databases like KEGG and Reactome [111]. Pathfinding algorithms can detect optimal relationships among biomedical entities by identifying the shortest or longest path connecting two nodes. In CKD, the Minimum Weight Spanning Tree was applied to find the minimum spanning tree of cytokine relationships, highlighting IL‐1β’s central role in CKD inflammation [112]. Each methodology reveals distinct properties of network nodes. Figure 2 illustrates key features of a specific network based on the type of analysis applied.

### 3.5 Biomarker Selection

Identifying biomarkers of disease development and progression requires appropriate strategies that can shift through the high dimensionality of omics datasets to pinpoint the most relevant molecular features distinguishing between disease groups. This can be achieved through, for example, using variable filtering techniques based on independent relevance measures (e.g., variance, correlation), supervised and unsupervised models, or network analysis techniques, as outlined above.

Sparsity‐inducing models are among the most popular for high‐dimensional, multicollinear omics data, optimizing model performance while selecting biologically meaningful features. The lasso [113] uses an L1‐norm penalty to enforce sparsity by shrinking less important model coefficients to zero, selecting only the most relevant variables. Lasso regression has been applied in CKD and DN studies to select metabolomics and transcriptomics features as potential biomarkers [80–82, 87, 100]. The lasso method has also been combined with RF, which ranks feature importance, and the Boruta algorithm, a RF‐based wrapper method, to identify candidate genes associated with CKD‐ICM [85].

## 4 Translational Value of Omics‐Derived Findings

### 4.1 Validation Studies

Biological validation is essential after identifying diagnostic and prognostic markers through ML, yet many studies lack verification in external cohorts or experiments. This step is crucial to confirm clinical relevance. Most CKD ML studies target disease progression [114], risk prediction [115], biomarker discovery, and pathophysiology [116], often using EHR or, more recently, omics data. Typically, models are developed with a training dataset and evaluated on a testing dataset, often from single‐center data (internal validation). External validation with independent data is usually lacking and noted as a study limitation. Exceptionally, a diagnostic model for CKD‐ICM based on 13 candidate genes was validated both internally and externally, using different GEO datasets than the ones used to develop the model [85]. Xie et al. identified differentially expressed genes associated with neutrophil extracellular traps (NETs) in human DKD kidney biopsy datasets and validated the results using single‐nucleus (sn) RNA sequencing datasets [116]. Chen et al. performed multi‐omics data integration using an assay for transposase‐accessible chromatin‐seq and scRNA‐seq GEO datasets and scRNA‐seq in human kidneys, to evaluate the transcriptional dynamics in fibrotic kidneys across mouse and human [117]. Additionally, the Nuclear Factor 1 X‐type (NFIX) promoting the apoptosis‐related gene Interferon alpha‐inducible protein 27 (IFI27) expression found by multimodal data was validated in an in vivo Adenoma‐Associated Virus (AAV)‐injected and unilateral ureteral obstruction mouse model, showing that the IFI27 level within the kidney was associated with fibrosis [117]. Also, Zhong et al. validated four diagnostic markers for DKD (tenascin C, peroxidasin, tissue inhibitor metalloproteinases 1, and tropomyosinin), using a human transcriptome GEO dataset and in a mouse model of DM and DKD [81]. Despite the surge in CKD‐related‐omics studies, external validation remains limited. Only through interdisciplinary collaboration, standardized preclinical models, and accessible high‐quality ‐omics data can we ensure societal return on investment and patient benefit.

### 4.2 AI‐Discovered Drugs

Following preclinical validation, ML‐derived biomarkers must be assessed in clinical trials to confirm their safety, efficacy, and clinical utility, ensuring they reliably predict or monitor disease progression and treatment response for patient care. Jayatunga et al. recently evaluated the clinical trial success rates of AI‐discovered drugs [118]. Their analysis was based on 75 clinically tested compounds since 2015 when screening the pipelines from over 100 AI‐native biotech companies. AI‐discovered drugs included AI‐discovered small molecules, biologicals, and vaccines, AI‐repurposed drugs, as well as compounds addressing AI‐discovered drug targets. The phase I success rate of around 87% is impressive and higher than the industry standard. This number is however based on a small sample size with 21 of 24 compounds being successful in phase I trials. The number of AI‐discovered drugs with results from phase II clinical programs is even smaller with four out of 10 compounds (40%) being considered successful, which is in the range of success rates for the pharmaceutical industry in general. Around half of the studies of AI‐discovered drugs were conducted in the field of oncology. This is in line with data consolidated by Andrii Buvailo who investigated the pipeline programs of some of the largest AI‐native biotech companies over the last 5 years [https://www.biopharmatrend.com/ai‐drug‐discovery‐pipeline/]. There are a few AI‐native biotechs with development programs in the field of nephrology including Insilico Medicine, BenevolentAI, Schrödinger, and Valo Health. BenevolentAI has one co‐development program together with AstraZeneca ongoing in the context of chronic kidney disease, however only being in the discovery phase so far. Insilico Medicine is testing a Traf2‐ and Nck‐interacting protein kinase (TNIK) inhibitor in the context of kidney fibrosis as well as the prolyl hydroxylases (PHD)1/2 inhibitor for anemia in chronic kidney disease. Schrödinger is running a co‐development program together with Bristol Myers Squibb with a focus on renal cell carcinoma. None of these compounds have so far progressed to clinical stage II testing, however. Delta4 used computational drug repositioning to identify clopidogrel as an attractive therapeutic option for the treatment of focal segmental glomerulosclerosis and proposed a phase II trial as a productive next step [119]. The most mature program in the context of kidney disease is a sphingosine‐1‐phosphate (S1P)1 agonist that is currently tested in phase II trials in heart failure and acute kidney injury by Valo Health. The provided data from Jayatunga et al. on success rates of AI‐based drugs across all therapeutic areas are promising although still being based on a small sample size. This holds even more true when we only consider AI‐discovered drugs in the area of nephrology where only very few compounds have yet entered clinical testing.

## 5 Economics and Cost‐Effectiveness of AI in CKD Management

### 5.1 Cost‐Effectiveness of AI in Healthcare

AI is increasingly being recognized for its potential to enhance healthcare outcomes while reducing costs, particularly in the management of chronic diseases like CKD. Given the growing burden of CKD worldwide, the use of AI for early detection, risk stratification, and personalized treatment offers significant opportunities for cost savings. Wolff et al. highlight the importance of conducting detailed cost‐effectiveness studies to quantify the benefits of AI technologies in healthcare settings [120].

Early diagnosis and intervention through AI represent crucial economic advantages in CKD management. By leveraging AI's predictive capabilities, healthcare systems can identify at‐risk patients before they develop severe symptoms, potentially saving millions in treatment costs [121, 122]. For instance, detecting CKD in its early stages allows for lifestyle modifications and preventive treatments that cost significantly less than late‐stage interventions like dialysis or transplantation. The economic impact is substantial, as early‐stage CKD management costs are typically a small fraction of the extensive costs associated with dialysis treatment. AI's predictive models have demonstrated particular effectiveness in early‐stage CKD, where they can prevent progression to end‐stage renal disease (ESRD), which is associated with the highest treatment costs [123]. Studies have shown that AI‐driven early detection and intervention systems can significantly reduce hospitalization rates through real‐time monitoring and personalized interventions [124], making a strong economic case for preventive care in CKD management.

#### 5.1.1 Cost‐Saving Potential of AI in CKD

AI shows strong potential to reduce CKD management costs by enhancing diagnostic and therapeutic processes. Through large dataset analysis, ML models identify early‐stage CKD, which is key to slowing disease progression and reducing reliance on costly treatments such as dialysis or transplantation [121, 123]. This is significant, given the high treatment costs; for instance, Medicare's CKD spending in the United States reached nearly USD 100 billion in 2015 [125].

Predictive AI models help forecast CKD progression, enabling timely treatment adjustments that prevent costly hospitalizations [122, 126]. This is crucial, especially given the disparities in CKD‐related disability‐adjusted life‐years (DALYs) across countries with lower socio‐demographic indexes, showing AI's role in alleviating CKD's economic burden [127].

Additionally, AI minimizes medical errors and improves resource allocation by identifying high‐risk CKD patients who need prioritized care. Studies indicate that AI can reduce CKD hospitalizations by up to 30% through real‐time monitoring and personalized care [124]. Integrating AI with telemedicine further enhances patient monitoring, reducing in‐person visit frequency and easing outpatient care costs and provider workload [128].

#### 5.1.2 Comparing Diagnostic AI and Therapeutic AI

The cost‐effectiveness of AI in CKD management varies by application. AI‐driven diagnostic tools, particularly those analyzing medical data and images, are highly accurate but most cost‐effective when leading to actionable results. For example, AI can analyze clinical data to detect CKD progression patterns earlier than traditional methods, enabling interventions that may lower future treatment costs [121, 123].

AI in treatment management offers greater long‐term savings. AI‐driven models that adjust therapies based on patient data optimize medication dosages, reduce side effects, and lower progression risks [126]. Based on their systematic review, Vithlani et al. found that 13 out of 21 studies (62%) demonstrated AI‐based healthcare interventions to be cost‐effective or cost‐saving compared to standard care, though most studies had significant methodological limitations and lacked transparent reporting about implementation costs [129].

### 5.2 Health Economic Models Versus Cost‐Effectiveness Models

Health economic and cost‐effectiveness models are vital for evaluating the financial impact of AI in CKD management, guiding policymakers and healthcare providers in resource allocation for AI infrastructure.

#### 5.2.1 Health Economic Models in CKD and AI

Health economic models consider both direct (e.g., equipment, training) and indirect costs (e.g., hospitalizations, long‐term care) of AI technologies. Wolff et al. stress these models' importance in understanding CKD's economic burden and AI's role in reducing it through early diagnosis and personalized care [120]. AI‐driven decision support systems help optimize healthcare resources, focusing on high‐risk patients to cut unnecessary treatments and readmissions, major cost contributors in CKD [130]. By integrating ERH and omics data, AI improves risk assessment, leading to more cost‐effective decisions [124]. These models also assess AI scalability, providing a full view of its economic viability and supporting early AI adoption for long‐term savings [122, 129].

#### 5.2.2 Cost‐effectiveness Models in CKD and AI

Cost‐effectiveness models compare AI intervention costs and outcomes to traditional approaches, often using Quality‐Adjusted Life Years (QALYs) to evaluate value [126]. In CKD, AI often incurs higher upfront costs but provides substantial long‐term savings by enhancing patient outcomes and reducing late‐stage interventions. For instance, Xie et al. found AI most cost‐effective in early‐stage CKD, preventing progression to costly kidney failure [123]. However, these models indicate that cost‐effectiveness depends on healthcare settings that can support AI infrastructure [121]. Additionally, AI improves QALYs by enabling timely, targeted treatments that delay CKD progression, enhancing both patient well‐being and long‐term cost savings [122, 130].

#### 5.2.3 Challenges in Modeling AI's Economic Impact

While valuable, these models face challenges. Wolff et al. note that traditional models struggle to capture AI's dynamic nature, as AI systems continuously adapt to new data, complicating long‐term predictions [120]. Furthermore, cost‐effectiveness models tend to emphasize short‐term outcomes, potentially overlooking AI's long‐term benefits in preventing complications [124, 130]. Additionally, ethical and legal considerations such as data privacy, consent, and liability carry economic implications [131, 132]. Future research should develop models that reflect AI's evolving capabilities and address these ethical complexities to enhance CKD management.

## 6 Future Directions for AI and Cost‐Effectiveness in CKD

This review outlines key steps for translating omics research into cost‐effective clinical strategies for CKD management. While integrating omics data with advanced ML offers clear benefits, limitations remain that need addressing to improve data and AI tool efficacy in CKD care. Below we highlight future directions and opportunities in AI for CKD care.

### 6.1 Data Availability

Despite various consortia creating valuable datasets, access is often hindered by inactive databases or fragmented data sources. Moreover, many CKD‐specific omics databases do not provide raw or preprocessed data, limiting research and analysis, as they mainly support validation by allowing disease‐related molecular signature assessments [9, 47, 48]. General repositories offer CKD samples with raw or preprocessed omics data but often restrict access to clinical data. Platforms like the Registry of Research Data Repositories and Database Commons centralize resources for reuse, while project websites control data openness [133, 134, 135]. The PerMediK network addresses these challenges, promoting data sharing, integration, and analysis for ML‐driven personalized medicine in CKD [136].

### 6.2 Validation Studies

Future efforts should focus on validating ML findings with independent datasets to improve generalizability and clinical efficacy. Biomarker discoveries must also be followed by deeper investigations into pathological mechanisms using diverse experimental models. The International Society of Nephrology's 2023 guidance on preclinical animal models in translational nephrology underscores the importance of model standardization [137]. Although primarily intended for drug development, these guidelines also aid in selecting models that closely mimic human CKD mechanisms. Additionally, the 3R principles (Replacement, Reduction, Refinement) should guide the process, encouraging alternatives like organs‐on‐a‐chip or organoids where possible [138, 139].

### 6.3 Advancing AI Cost‐Effectiveness in CKD

To achieve cost‐effective AI in CKD care, AI must deliver actionable insights for timely interventions and prevent costly complications. While initial investments in infrastructure and skilled personnel are substantial, AI's ability to reduce hospitalizations, improve diagnostic accuracy, and enhance patient adherence presents clear long‐term economic benefits. Future research should increase the application and development of AI algorithms for CKD diagnosis and treatment through multi‐omics data integration, facilitating targeted preventive interventions that contribute to overall cost savings [140]. ML can identify high‐risk patients for earlier interventions and personalized care [121, 129]. Furthermore, advances in cloud computing and edge AI support real‐time decision‐making by aggregating multiple sources of data [124, 126, 130].

### 6.4 Policy Recommendations for AI Adoption in CKD Care

Supportive policies are essential for AI's widespread adoption in CKD care. Regulatory clarity, data privacy, and standardization are critical challenges [120]. Policymakers should establish guidelines to ensure AI accuracy, safety, and data security. Incentives, such as grants and partnerships, can help low‐resource institutions offset initial AI costs, encouraging broader adoption [130]. Collaboration among governments, healthcare providers, and tech firms could produce tailored, cost‐effective AI solutions [122].

### 6.5 Addressing Research Gaps and Challenges

Standardization and explainability are major challenges for AI in CKD management. Differences in data quality, demographics, and infrastructure affect AI performance, reducing generalizability [124]. Future research should focus on adaptable models that maintain accuracy across diverse settings. Additionally, enhancing AI explainability is crucial for provider and patient trust, requiring transparency in algorithms [121, 141].

### 6.6 Expanding the AI's Role in Preventive CKD Care

AI's future in CKD may extend to preventive strategies by identifying early signs in asymptomatic patients, supporting lifestyle modifications, and early interventions [129, 130]. Integration with health monitoring tech could allow AI to predict health deterioration, reducing the need for intensive interventions and lowering long‐term CKD treatment costs [122, 124].

### 6.7 Multidisciplinary Collaboration

The successful integration of AI into CKD management holds the promise of more efficient, effective, and economically sustainable care, balancing innovation with healthcare realities. The future of AI in CKD benefits from multidisciplinary collaboration among data scientists, clinicians, bioinformaticians, and policymakers.

## Conflicts of Interest

The authors have declared no conflict of interest. M.A.J.C. is an employee of Mosaiques Diagnostics. M.L. and P.P. are employees of Delta 4 GmbH.

## Data Availability

The author has provided the required Data Availability Statement, and if applicable, included functional and accurate links to said data therein.
